# Supplementary material for: Understanding Parental Adherence to Early Childhood Domestic Injury Prevention: A Cross-Cultural Test of the Integrated Behavior–Change Model
Source: Behav Sci (Basel). 2024 Aug 12;14(8):701. doi: 10.3390/bs14080701 (PMC11351157; doi:10.3390/bs14080701)
Supplement: Supplementary file 1 [file behavsci-14-00701-s001.zip › behavsci-3092056-supplementary.pdf]

Table S1. Scales and items for this study (Supplementary Online Material).

**Scale**	**Items**
Health Care Climate Questionnaire	Anchors: 1 = strongly disagree, 7 = strongly agree.<br>Stem: To prevent my grandchild from domestic injuries…<br>1. I feel that other family members have provided me with choices and options.<br>2. I feel understood by other family members.<br>3. Other family members convey confidence to do well at my job<br>4. Other family members encourage me to ask questions.<br>5. Other family members listen to how I would like to do things.<br>6. Other family members try to understand how I see things before suggesting a new way to do things.
Treatment Self-Regulation Questionnaire for Domestic Injury Prevention	Anchors: 1 = not true at all, 7 = very true.<br>Stem: I want to prevent my grandchildren from domestic injuries because…<br><u>Autonomous motivation</u><br>1. I feel that I want to take responsibility for my grandchild(ren)'s health.<br>2. Because I personally believe it is the best thing for my grandchild(ren)'s health.<br>3. Because I have carefully thought about it and believe it is very important for many aspects of my life.<br>4. Because it is an important choice that I really want to make.<br>5. Because it is consistent with my life goals.<br>6. Because it is very important for being as healthy as possible.
Theory of Planned Behavior Questionnaire	Stem: Reducing the likelihood of childhood domestic injury in the forthcoming month is something that…<br>Anchors: 1 = strongly disagree, 7 = strongly agree<br><u>Intention</u><br>1. I intend to do.<br>2. I will try to put great effort into doing.<br>3. I plan to do.<br><u>Social Norms</u><br>4. Most people who are important to me think that I should do.<br>5. People expect me to do.<br>6. Important people in my life would approve of me doing.<br><u>Perceived Behavior Control</u><br>7. Is possible for me to do.<br>8. I could do if I wanted to.<br>9. I have complete control over how to do.<br>10. Mostly up to me whether or not to do.<br>11. Is easy for me to do.<br>Attitude<br>Stem: Reducing the likelihood of childhood domestic injury in the forthcoming month is something that is…<br>12. Anchor: 1 = Worthless, 7 = Valuable<br>13. Anchor: 1 = Harmful, 7 = Beneficial<br>14. Anchor: 1 = Unpleasant, 7 = Pleasant<br>15. Anchor: 1 = Unenjoyable, 7 = Enjoyable<br>16. Anchor: 1 = Bad, 7 = Good
Self-Reported Domestic Injury Prevention Adherence Scale	<u>Frequency</u><br>Anchors: 1 = Never, 7 = Very often.

	1. How often do you take measures against domestic injury for your grandchild(ren)?<br>Effort<br>Anchors: 1 = Minimum effort, 7 = Maximum effort.<br>Stem:<br>2. How much effort do you put into preventing domestic injury for your grandchild(ren)?
